# A joint convolutional-recurrent neural network with an attention mechanism for detecting intracranial hemorrhage on noncontrast head CT

**DOI:** 10.1038/s41598-022-05872-x

**Published:** 2022-02-08

**Authors:** Deniz Alis, Ceren Alis, Mert Yergin, Cagdas Topel, Ozan Asmakutlu, Omer Bagcilar, Yeseren Deniz Senli, Ahmet Ustundag, Vefa Salt, Sebahat Nacar Dogan, Murat Velioglu, Hakan Hatem Selcuk, Batuhan Kara, Caner Ozer, Ilkay Oksuz, Osman Kizilkilic, Ercan Karaarslan

**Affiliations:** 1 Radiology Department, Acibadem Mehmet Ali Aydinlar University School of Medicine, Istanbul, Turkey; 2 Neurology Department, Istanbul Istinye State Hospital, Istanbul, Turkey; 3 Department of Software Engineering and Applied Sciences, Bahcesehir University, Istanbul, Turkey; 4 Department of Radiology, Istanbul Mehmet Akif Ersoy Thoracic and Cardiovascular Surgery Training and Research Hospital, Halkali, Istanbul, Turkey; 5 Radiology Department, Istanbul Silivri State Hospital, Istanbul, Turkey; 6 Radiology Department, Cerrahpaşa Medical Faculty, Istanbul University-Cerrahpasa, Istanbul, Turkey; 7 Radiology Department, Acibadem Atakent Hospital, Istanbul, Turkey; 8 Radiology Department, Istanbul Fatih Sultan Mehmet Training and Research Hospital, Istanbul, Turkey; 9 Radiology Department, Istanbul Bakırköy Sadi Konuk Training and Research Hospital, Istanbul, Turkey; 10 Computer Engineering Department, Istanbul Technical University, Istanbul, Turkey

**Keywords:** Stroke, Computer science

## Abstract

To investigate the performance of a joint convolutional neural networks-recurrent neural networks (CNN-RNN) using an attention mechanism in identifying and classifying intracranial hemorrhage (ICH) on a large multi-center dataset; to test its performance in a prospective independent sample consisting of consecutive real-world patients. All consecutive patients who underwent emergency non-contrast-enhanced head CT in five different centers were retrospectively gathered. Five neuroradiologists created the ground-truth labels. The development dataset was divided into the training and validation set. After the development phase, we integrated the deep learning model into an independent center’s PACS environment for over six months for assessing the performance in a real clinical setting. Three radiologists created the ground-truth labels of the testing set with a majority voting. A total of 55,179 head CT scans of 48,070 patients, 28,253 men (58.77%), with a mean age of 53.84 ± 17.64 years (range 18–89) were enrolled in the study. The validation sample comprised 5211 head CT scans, with 991 being annotated as ICH-positive. The model's binary accuracy, sensitivity, and specificity on the validation set were 99.41%, 99.70%, and 98.91, respectively. During the prospective implementation, the model yielded an accuracy of 96.02% on 452 head CT scans with an average prediction time of 45 ± 8 s. The joint CNN-RNN model with an attention mechanism yielded excellent diagnostic accuracy in assessing ICH and its subtypes on a large-scale sample. The model was seamlessly integrated into the radiology workflow. Though slightly decreased performance, it provided decisions on the sample of consecutive real-world patients within a minute.

## Introduction

Intracranial hemorrhage (ICH) is a life-threatening condition with high mortality rates^1,2^. ICH might occur spontaneously or due to head trauma, and regardless of the underlying cause, non-contrast head CT is the method of choice for the radiological diagnosis^3^. The rapid and accurate diagnosis is crucial as the clinical deterioration often occurs within the first few hours after ICH onset. Furthermore, there is a need for precise estimation of ICH subtypes, namely intraparenchymal hemorrhage (IPH), intra-ventricular (IVH), subarachnoid (SAH), subdural, (SDH), and epidural hemorrhage (EDH), as the type of ICH closely relates with the prognosis and treatment options^4^. However, delays in the report turn-around time are an issue of concern^5^. Expert radiologist shortage is another source of the problem, often being compensated by the residents or non-radiologist clinicians in the emergency settings, particularly after work hours. The aforementioned issues inevitably lead to misdiagnosis and late diagnosis^6–8^.

Before the deep learning (DL) era, researchers mainly used traditional machine learning methods combined with human-engineered features for automated ICH detection on non-contrast CT^9^. Unfortunately, traditional methods' diagnostic performances have not reached acceptable levels for integration into the clinical workflows^10^. The last decade witnessed rapid developments in computer vision, and convolutional neural networks (CNN), a kind of DL method, have played the dominant role in these advancements^11^. Unlike traditional machine learning, DL can simultaneously identify the best features for a task at hand and performs these tasks, such as classification, object detection, and segmentation. Besides, its scalability to data size is a major advantage as large datasets significantly boost its performance^11^. Several preceding studies have demonstrated DL's yields in identifying ICH on non-contrast head CT scans, which encourages using DL in clinical practice^12–14^. Nevertheless, it is well-known that DL models' performance should be explored on unseen test data, preferentially on an external sample, to precisely uncover the models' generalizability^15^. However, only a few studies investigated the generalizability of DL on multi-center large-scale datasets^13,14,16^ or implemented the DL models into the clinical workflow^12–14,17,18^.

The present study used a novel DL architecture, a joint CNN recurrent neural network (RNN) with an attention mechanism, to detect and subcategorize ICH on non-contrast head CT scans on a large-scale multi-institutional sample. The model's decision was explored by applying a novel approach, the NormGrad method^19^, an advancement over its antecedents, to ameliorate DL’s black-box nature. We also evaluated the proposed model's performance on prospectively obtained non-contrast head CT examinations ordered from the emergency department for over six months in a different center.

## Materials and methods

**Figure 1:**
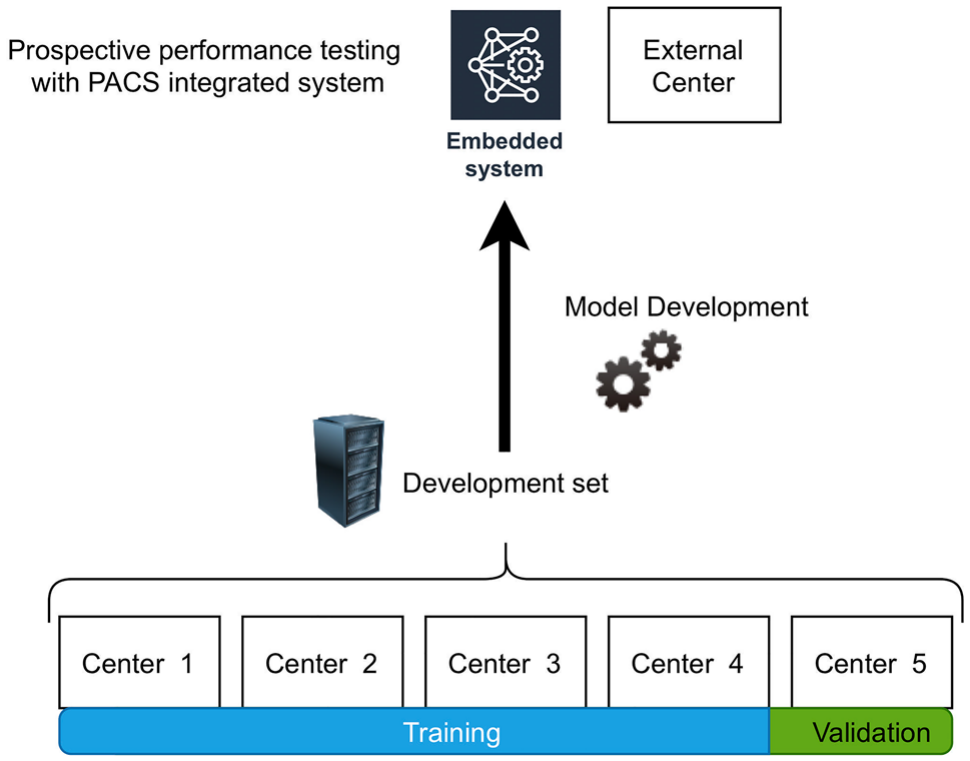
The flowchart of the study (The image was created by the authors using Microsoft PowerPoint v16). We obtained consecutive non-contrast-enhanced CT scans referred from the emergency service in five different tertiary care centers. Data from four centers were used as the training, and the remaining were used as the validation data. The final model was integrated into the Picture archiving and communication system (PACS) on a dedicated embedded unit. The model's performance was assessed on consecutive emergency non-contrast head CT scans for over six months. The diagnostic and inference performance of the system was documented.

This multi-center study was carried out between January 2015 and December 2020. Acibadem Mehmet Ali Aydinlar University's ethics committee approved the study. For the retrospective study phase, the ethics committee waived the need for informed consent. For the clinical implementation, informed consent was obtained from the participants. All consecutive adult patients who underwent non-contrast-enhanced head CT referred from the five tertiary centers' emergency services were enrolled in the present study. Head CT scans of patients < 18 years of age were excluded from the study. All remaining scans, including the examinations with intra- or extra-axial mass lesions, post-operative examinations, and examinations with severe motion or metal artifacts, were included to gather a representative dataset of the real clinical setting. The head CT with chronic hemorrhages or hemorrhagic mass lesions was accepted as ICH positive. All examinations were anonymized before the analysis. The study sample (henceforth named as the development set) was partitioned into training and validation datasets. Four of the five centers' data constituted the training, and the remaining one constituted the validation set. Figure 1 shows the flowchart of the study.

### Ground-truth annotations

Five neuroradiologists with over ten years of neuroradiology experience from each center examined the recruited images. The neuroradiologists were free to assess all the available clinical and radiological data during the evaluation. Briefly, the neuroradiologist evaluated the images for the presence of hemorrhage, if it exists, its subtypes as IPH, IVH, SDH, EDH, and SAH. All the annotations were performed on a slice basis. The slices of a post-operative examination were labeled as ICH-positive if it contained hemorrhage apart from the post-operative changes (i.e., operation material). The slices with mass lesion (i.e., primary or secondary tumors), acute or chronic ischemic lesion, or metallic instruments were annotated as ICH-negative if they did not contain any pixel with hemorrhage. All CT images were resampled with a slice thickness of 5 mm before the labeling.

The annotation quality of the dataset is of vital importance for the performance of DL models. However, given the high number of examinations, it was impossible to re-evaluate all the images using another reader to ensure correctness. In such large image sets, the best practice is to ensure the validity of the validation and tests to precisely estimate the performance and tune the model as the DL models is quite robust to non-systematic errors in the training set (e.g., skipping the slice with hemorrhage during the annotation or inadvertently mistaken labeling ICH subtypes)^20^. Thus, each examination in the validation set was cross-validated by two other neuroradiologists in a random order, and the majority voting was used to determine the final ground-truth labels of an examination per-slice basis.

### The joint CNN-RNN model with an attention mechanism

**Figure 2:**
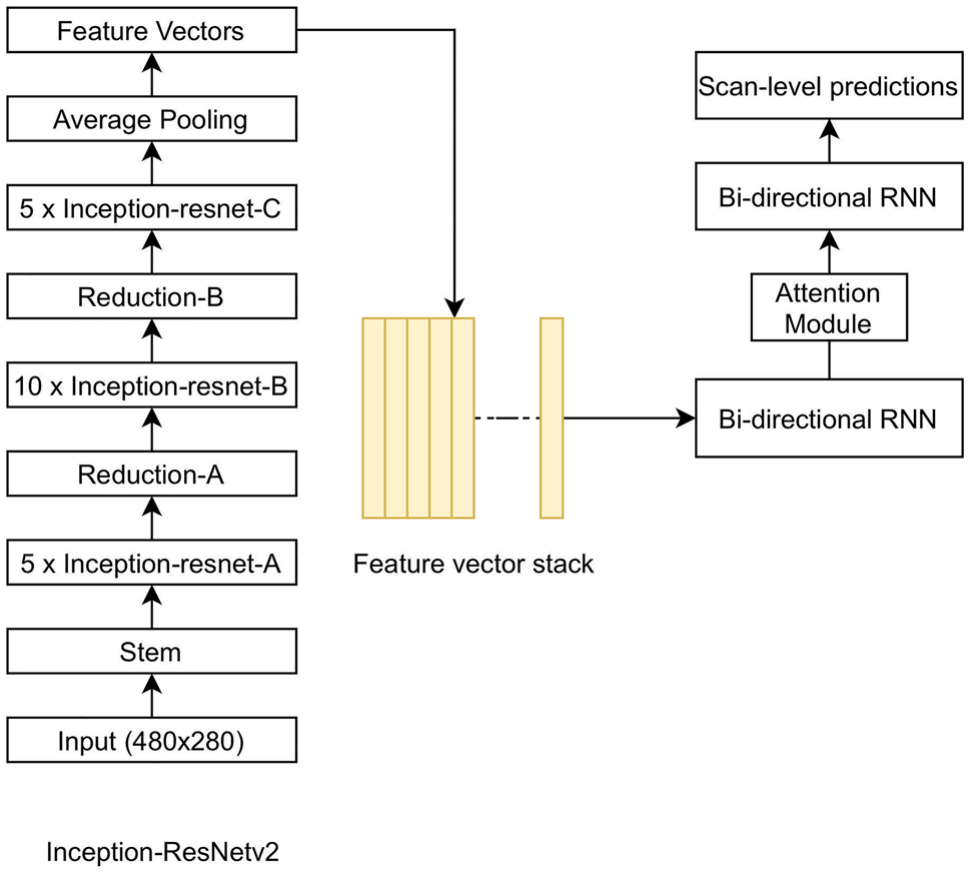
A diagram showing the joint convolutional neural network (CNN)-recurrent neural network (RNN) with an attention mechanism (The image was created by the authors using Microsoft PowerPoint v16). We used InceptionResNetV2 as the feature extractor with its top predictions layer removed. The extracted features were stacked per scan and fed into the bi-directional RNN. We placed an attention layer between two layers of the RNN, which facilitates RNN to focus on the most relevant slices to identify ICH and its subtypes.

All DL experiments were conducted using a DL library, TensorFlow (Tensorflow 2.4 Google LLC, Mountain View, CA), on a custom-built workstation equipped with a 24 GB graphical processing unit. The present work used InceptionResNetV2 as the base network for extracting the most relevant features from the images^21^. The CNN model had 55,873,736 parameters with a depth of 572 layers. The extracted images were fed the bi-directional RNN with an interspersed attention layer. This structure enabled the model to convey the information between the slices of an examination making its final prediction^22^. The attention mechanism facilitates bi-directional RNN in focusing on the most relevant data for the task at hand^23^. The average training time for the training was 37 days. The model was trained with the following parameters: The loss was the binary cross-entropy^24^ for each ICH class; the optimizer was adaptive moment estimation (Adam)^25^; the learning rate was set at 1e-3 with exponential decay of 0.96 per epoch^26^. Figure 2 illustrates the joint CNN-RNN with the attention mechanism.

Head CT images were fed into the networks using three different windowing settings (WL/WW: 50–100, 50–130, and 150–300) to accentuate contrast differences between the background and ICH. In addition, several on-the-fly typical image pre-processing operations were performed on the images before feeding them into the network: (1) intensity normalization within 0–1; (2) Resizing the images into the shape of 480 × 480; and (3) data augmentations including cropping, rotation, flipping, and elastic deformations.

### Model interpretability

We implemented a modified version of Gradient-based class activation maps (Grad-CAM), a well-established saliency map generating method, NormGrad, for highlighting how the model makes its decision for the given task. NormGrad calculates the outer product between each vectorized component of activation maps and gradients and uses Frobenius Norm, preserving the information in exhibited regions^19^. We hypothesize that NormGrad would yield more delicate activation maps than the Grad-CAM; thus, it would be much more amenable to be used in medical imaging tasks where the pathology often occupies a much smaller area than the background. A four-point Likert-scale (four-points: excellent quality; three-points: good quality; two-points acceptable quality; and one-point: bad quality) was used to assess the quality of the saliency maps subjectively. The same five neuroradiologists independently reviewed randomly sampled 2500 slices of different scans containing at least one of the ICH subtypes and scored the quality of NormGrad and Grad-CAM generated saliency maps slice-basis. The observers were blinded to the method while evaluating the saliency maps. The scores of the observers were averaged to provide the final quality scores of the attention maps.

### Clinical implementation

To assess the proposed model's generalizability on the independent external dataset and explore the feasibility of implementing DL models into the clinical environment, we embedded the developed DL model into a hardware module specially designed for the inference (Jetson NVIDIA). In brief, this module is connected to the Picture archiving and communicating system (PACS) of an external tertiary care center. The head CT examinations were automatically queried and retrieved from the PACS using the relevant series description. The embedded DL model made the predictions over the images and gave its final decision (i.e., ICH-positive or ICH-negative, and ICH subtype) per scan. Three radiologists with over 25, 15, and 8 years of head CT experience who were blinded to the model's decision during the annotation process assigned each scan's final diagnosis on a scan level (i.e., the presence of hemorrhage, and if present, its subtype); the majority voting was used to create the ground-truth annotations on a scan level.

### Statistical analyses

Statistical analysis was performed using Scipy library v1.5.4 of Python programming language (“https://docs.scipy.org”). All performance metrics were calculated and presented on a scan basis for clarity. The primary metric for investigating a model's performance was diagnostic accuracy accepting the ground-truth annotations as the reference. Other metrics used for assessing models' performance were the sensitivity, specificity, AUC, and F1-measure. For the clinical implementation phase, we also evaluated the inference time. The Mann–Whitney U test was used to compare NormGrad and Grad-CAM's subjective quality for delineating the pathology. A *P* value < 5% was considered as a statistically significant result.

### Ethical statement and consent to participate

All procedures performed in studies involving human participants were in accordance with the ethical standards of the institutional and/or national research committee and with the 1964 Helsinki declaration and its later amendments or comparable ethical standards. Acibadem Mehmet Ali Aydinlar University's ethics committee approved the study. For the retrospective study phase, the ethics committee waived the need for informed consent. For the clinical implementation, informed consent was obtained from the participants.

## Results

A total of 55,179 head CT scans of 48,070 patients, 28,253 men (58.77%), with a mean age of 53.84 ± 17.64 years (range 18–89) were enrolled in the study. There were 15,733 ICH-positive scans (28.51%), while the remaining 39,446 (71.49%) examination was ICH-negative. The training sample comprised 49,968 head CT scans with 14,742 was annotated as ICH-positive by the neuroradiologists on the scan level. The validation sample comprised 5211 head CT scans with 991 (19.01%) was annotated as ICH-positive by the neuroradiologists on the scan level. There were 12,403 ICH-positive slices in the validation sample, whereas the number of ICH-negative slices was 165,843. Further details regarding the study sample are given in Table 1.

**Table 1:** Characteristics of the study sample.

Variables	Study sample (n = 48,070)	Training (n = 43,460)	Validation (n = 4610)	Testing (n = 380)
**Age**	54 (IQR, 43–65)	54 (IQR, 43–65)	53 (IQR, 40–60)	48 (IQR, 36–57)
**Gender (Male)**	28,253 (58.77%)	25,079 (57.70%)	3174 (68.85%)	
**ICH-positive patients**	13,224 (27.50%)	9226 (21.22%)	612 (13.27%)	130 (34.21%)
**CT examinations**	55,179	49,968	5211	452
***ICH (binary)***	15,733 (28.51%)	14,742 (29.50%)	991 (19.01%)	167 (36.9%)
*IPH*	10,080 (18.26%)	9422 (18.85%)	658 (12.62%)	86 (19.02%)
*IVH*	5963 (10.78%)	5535 (11.07%)	418 (8.02%)	38 (8.4%)
*SAH*	9555 (17.31%)	8955 (17.92%)	600 (11.51%)	48 (10.61%)
*SDH*	7473 (13.54%)	7022 (14.05%)	451 (8.65%)	76 (16.81%)
*EDH*	1237 (2.24%)	1116 (2.33%)	71 (1.35%)	14 (3.1%)
**Total CT slices**	2,255,271	2,255,271	212,873	–
*ICH-positive*	188,067 (8.33%)	175,664 (7.78%)	12,403 (5.82%)	–
*ICH-negative*	2,067,204 (91.67%)	2,079,607 (92.22%)	200.470 (6.18%)	–

*EDH epidural hemorrhage, *ICH* intracranial hemorrhage, *IPH* intra-parenchymal hemorrhage, *SAH* subarachnoid hemorrhage, *SDH* subdural hemorrhage.

The joint CNN-RNN with an attention mechanism yielded a diagnostic accuracy of 98.26% (95% CI 98.14–98.37%) with correctly classifying 49,101 out of 49,968 head CT scans on the training set. The sensitivity, specificity, and AUROC of the model on the training set was 97.72% (95% CI 97.58–97.85%), 98.49% (95% CI 98.42–98.55%), and 0.992 (95% CI 0.991–0.993), respectively. The model achieved a diagnostic accuracy of 99.41% (95% CI 99.51–99.84%) with correctly classifying 5180 out of 5211 head CT scans on the validation set. The sensitivity, specificity, and AUROC of the model on the validation set was 99.70% (95% CI 99.51–99.84%), 99.34% (95% CI 99.09–99.58%), and 0.998 (95% CI 0.998–0.999), respectively.

During the prospective clinical implementation phase, a total of 452 head CT scans of 380 patients were evaluated by the joint DL model for six months. During inference, the mean prediction time was 45 ± 8 s (range 35–59), including image transfer from the PACS to the embedded system in which DL models were implemented. Among 452 head CT scans, 167 had ICH, and the joint model correctly classified 434 scans in the clinical test set, equating an accuracy of 96.02 (95% CI 94.21–97.92). The other metrics regarding the model performance on the training, validation, and test sets are given in Table 2.

**Table 2:** Diagnostic performance of the unified CNN-RNN model on the training, validation, and testing sets.

ICH subtype	Diagnostic metrics					Confusion matrix		
	Sensitivity (95% CI)	Specificity (95% CI)	Precision (95% CI)	Accuracy (95% CI)	AUROC (95% CI)	Predictions		Ref. test
						Pos	Neg	
**Training**	97.72 (97.47–97.96)	98.49 (98.36–98.61)	96.45 (96.15–96.74)	98.26 (98.15–98.37	0.992 (0.991–0.993)	14,406	336	Pos
ICH-Binary						531	34,695	Neg
IPH	93.19 (92.67–93.69)	99.27 (99.18–99.37)	96.74 (96.37–97.10)	98.12 (98–98.24)	0.990 (0.989–0.991)	8780	642	Pos
						296	40,250	Neg
IVH	91.83 (91.11–92.55)	99.54 (98.47–99.60)	96.14 (95.62–96.66)	98.69 (98.53–98.78)	0.993 (0.991–0.994)	5083	452	Pos
						204	44,229	Neg
SAH	81.37 (80.56–82.17)	98.57 (98.45–98.68)	92.53 (91.95–93.11)	95.49 (95.30–95.66)	0.978 (0.976–0.980	7287	1668	Pos
						588	40,425	Neg
SDH	87.35 (86.57–88.13)	98.94 (98.84–99.04)	93.09 (92.48–93.71)	97.31 (97.17–97.48)	0.956 (0.954–0.958)	6134	888	Pos
						455	42,491	Neg
EDH	78.47 (76.11–80.822	98.94 (98.84–99.01)	63.28 (60.79–65.76)	98.43 (98.32–98.53)	0.988 (0.987–989)	915	251	Pos
						531	48,271	Neg
**Validation**	99.70 (99.35–100)	99.34 (99.09–99.58)	97.24 (96.24–98.25)	99.41 (99.19–99.61)	0.998 (0.996–0.999)	988	3	Pos
ICH-Binary						28	4192	Neg
IPH	95.90 (94.38–97.41)	99.28 (99.02–99.42)	95.03 (93.38–96.68)	98.85 (98.55–99.13)	0.998 (0.997–1)	631	27	Pos
						33	4520	Neg
IVH	95.22 (93.16–97.26)	99.56 (99.37–99.74)	94.99 (92.90–97.08)	99.21 (98.97–99.45)	0.998 (0.992–1)	398	20	Pos
						21	4772	Neg
SAH	84.50 (81.60–87.39)	99.09 (98.81–99.20)	92.35 (90.13–94.57)	97.41 (96.47–97.84)	0.991 (0.981–0.999)	507	93	Pos
						42	4569	Neg
SDH	91.13 (88.50–93.75)	99.33 (99.10–99.55)	92.78 (90.37–95.19)	98.62 (98.30–98.90)	0.974 (0.972–0.976)	411	40	Pos
						32	4728	Neg
EDH	74.61 (64.48–84.73)	98.83 (98.49–99.16)	51.56 (41.80–61.11)	98.50 (98.16–98.83)	0.980 (0.970–0.983)	53	18	Pos
						50	5090	Neg
**Testing**	96.41 (93.58–99.22)	95.79 (93.45–98.18)	93.06 (89.28–96.85)	96.02 (94.21–97.82	0.961 (0.941–0.982)	161	6	Pos
ICH-Binary						12	273	Neg
IPH	82.56 (74.53–90.57)	97.54 (95.95–99.12)	88.75 (81.83–95.67)	94.69 (92.62–96.75)	0.905 (0.888–0.925)	71	15	Pos
						9	357	Neg
IVH	86.84 (66.94–97.58)	98.31 (97.06–99.55)	82.50 (70.72–94.28)	97.35 (95.86–98.82)	0.925 (0.900–0.950)	33	5	Pos
						7	407	Neg
SAH	91.67 (83.84–99.48)	86.14 (82.76–89.5)	44 (34.27–53.73)	86.73 (83.69–89.85)	0.889 (0.863–0.925)	44	4	Pos
						56	348	Neg
SDH	88.16 (80.89–95.42)	90.16 (87.14–93.17)	64.42 (55.22–73.62)	89.82 (87.03–92.61)	0.891 (0.870–0.91)	67	9	Pos
						37	339	Neg
EDH	71.4 (47.72–95.07)	99.98 (99.84–1)	90.91 (73.92–100)	98.89 (97.15–99.9)	0.980 (0.96–1)	10	4	Pos
						1	437	Neg

*EDH epidural hemorrhage, *ICH* intracranial hemorrhage, *IPH* intra-parenchymal hemorrhage, *SAH* subarachnoid hemorrhage, *SDH* subdural hemorrhage.

**Figure 3:**
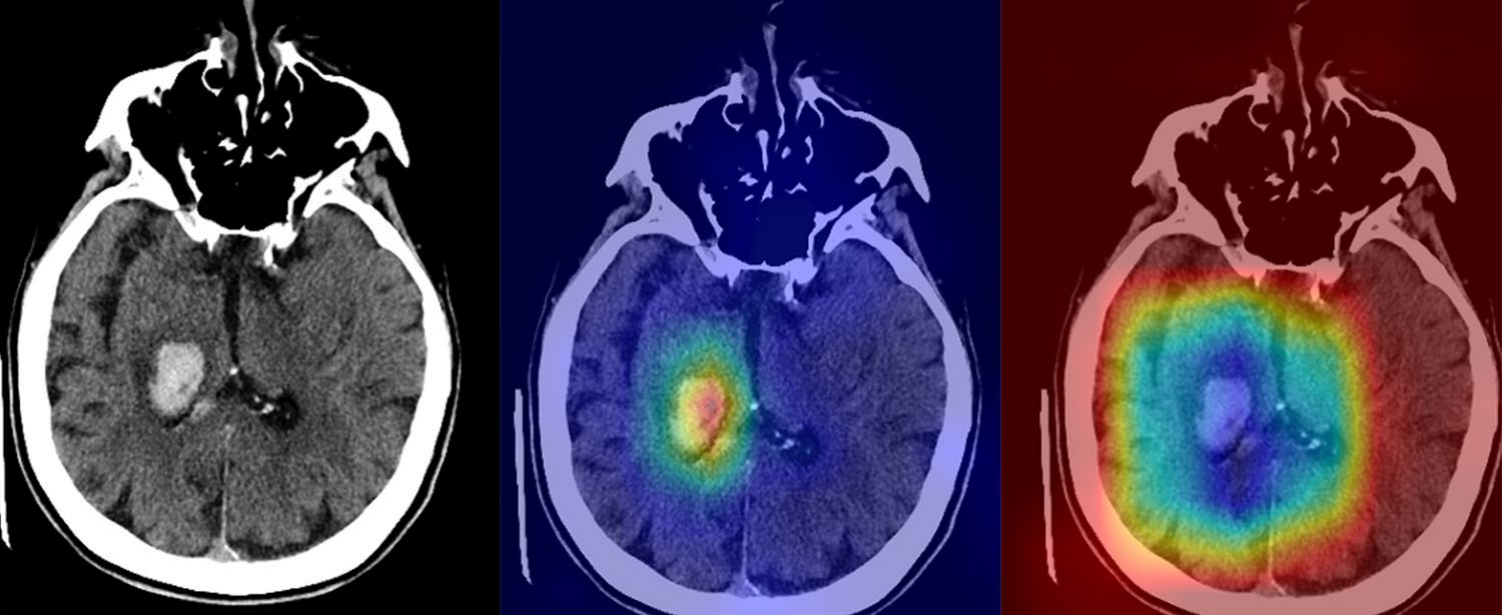
A 68-year-old female with known hypertension (The images were created by the authors using open-source software, Matplotlib v3.5, Python v3). A right thalamic hematoma extended into the adjacent ventricular system on a non-contrast head CT scan (right). NormGrad (middle) method generates more delicate saliency maps than Grad-CAM (left), highlighting the thalamic hematoma and its ventricular extension. The average quality scores were 3.6 points and 2 points for the NormGrad and Grad-CAM, respectively. Please note that the observers evaluated saliency maps with the same color spectrum, and the current color maps are adjusted for representative purposes.

**Figure 4:**
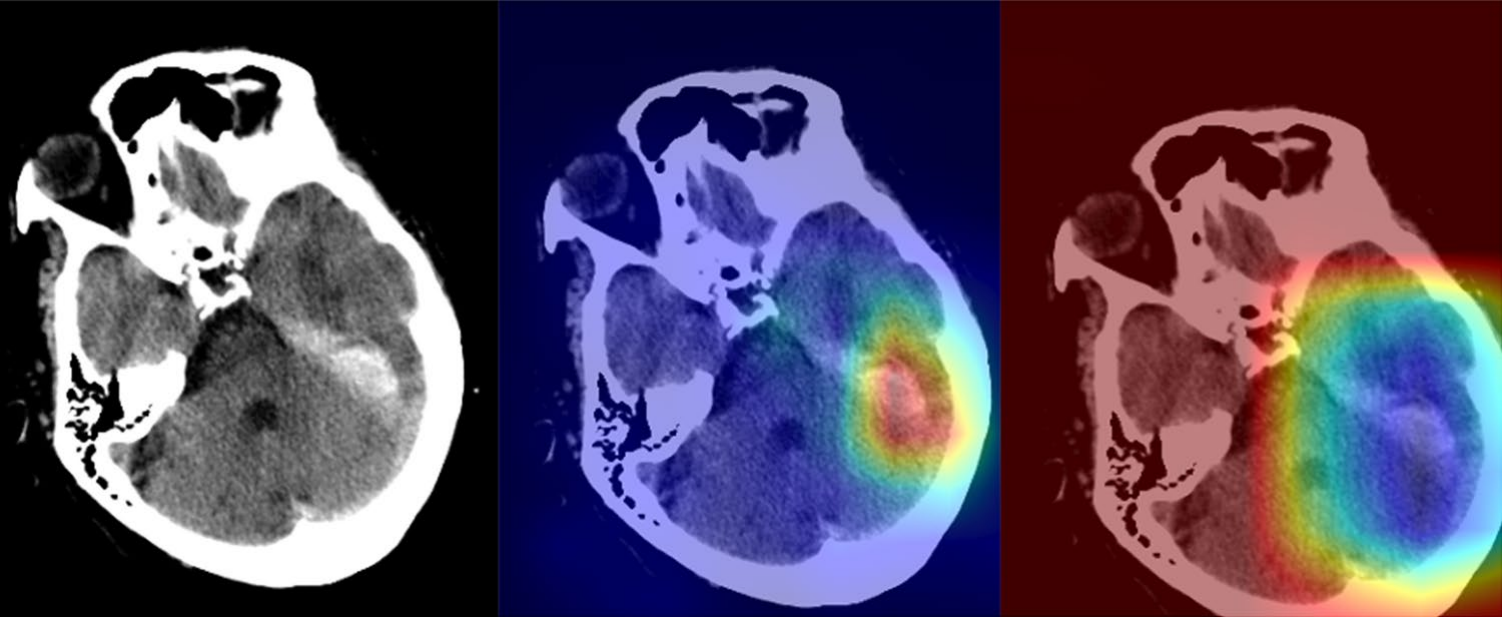
A 71-year-old man with a recent history of head trauma (The images were created by the authors using open-source software, Matplotlib v3.5, Python v3). Non-contrast head CT scan shows a subdural hematoma along the left tentorium cerebelli (right). NormGrad (middle) method generates finer saliency maps than Grad-CAM (left), highlighting the subdural hematoma. The average quality scores were 3.8 points and 1.8 points for the NormGrad and Grad-CAM, respectively. Please note that the observers evaluated saliency maps with the same color spectrum, and the current color maps are adjusted for representative purposes.

**Figure 5:**
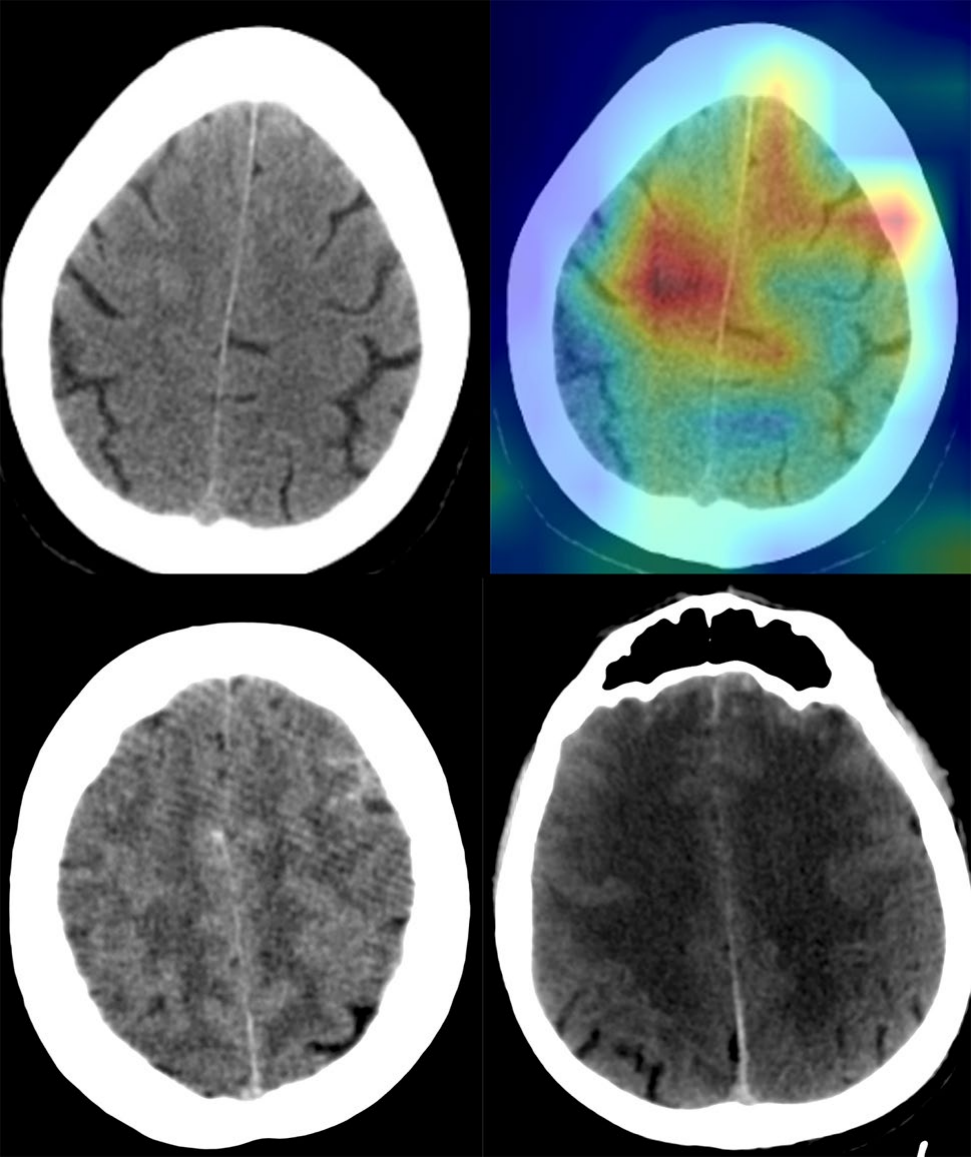
The presentative images of different patients in whom the model predictions were wrong (The images were created by the authors using open-source software, Matplotlib v3.5, Python v3). The original (the upper left) and corresponding normgrad images (the upper right) with a false-positive prediction are shown. In addition, the model overlooked the minor subarachnoid hemorrhage in the left frontal lobe (the lower left); the model missed the minor subarachnoid hemorrhage in the frontal lobe and subdural hemorrhage in the frontotemporal area (the lower right).

On the four-points scale, the average scan-based scores of the saliency maps generated by the NormGrad method were 3.3 ± 0.6 and 3.1 ± 0.4, whereas the Grad-CAM images yielded average scores of 2.1 ± 0.7 and 1.8 ± 0.5, for the observers. For both observers, the Mann–Whitney-U test showed that the NormGrad provided higher-quality decision maps than the Grad-Cam Method (*P* < 0.0001). Figures 3 and 4 show representative cases for the predictions of the model. Figure 5 shows several examples of incorrect predictions of the model.

## Discussion

### Key findings

The present work provided several relevant findings on the use of DL methods for assessing ICH on non-contrast-enhanced head CT: (1) The unified CNN-RNN model with the attention mechanism achieved an excellent diagnostic accuracy for identifying ICH on non-contrast-enhanced head CT, and good overall performance for categorizing its subtypes; (2) The use of NormGrad method instead of previously implemented Grad-CAM allows better saliency maps for explaining the model's decision, which might further improve the interpretability and obviate black-box nature of DL models; (3) The proposed model was seamlessly integrated into the PACS environment and showed a diagnostic accuracy of 96.02% on the independent external data during the clinical implementation phase, which encourages its use in the real clinical setting.

### Relevant work

Apart from several studies with a small sample size (i.e., less than 1000 samples)^18,27,28^, few studies investigated the utility of DL on a relatively large scale. Arbabshirani and colleagues implemented the CNN model for binary classification of ICH^14^. The authors reported relatively low diagnostic performance (AUC, 0.846) compared with the present work^14^. They integrated the DL model into clinical workflow and demonstrated the algorithm's benefits in prioritizing the routine head CT scans. The major weakness of their study appeared to be the lack of slice-based labels and subcategorization of ICH. We argue that the somewhat low performance might stem from the lack of slice-based annotations and a relatively simple CNN model. Chilamkurthy et al. applied DL to evaluate ICH on a large-scale national sample^13^. The authors trained their model on over three hundred thousand head CT scans and assessed its performance on a subset of their sample and independent external test set. They reported an AUC of 0.92 and 0.94 in detecting ICH on the validation and test sets, respectively, which were comparably lower than those obtained in the present work. The authors used a traditional ML method, random forest, instead of DL methods to aggregate the DL model's slice-based predictions. Additionally, they used radiology reports as the reference by leveraging natural language processing, which might result in erroneous annotations. We assume that these design choices might be accounted for the slightly lower performance.

In recent work, Cho et al. utilized cascaded DL models for ICH detection and lesion segmentation on a dataset derived from two different centers^29^. The first part of their cascaded network was used as the ICH identifier whilst the second part served to discriminate ICH subtypes and segment the lesions. The authors reached diagnostic accuracy of 98.28% on the validation set using five-fold cross-validation over the entire sample. However, the lack of an independent test set limited their study. Furthermore, it is well-known that the validation set should not be used as the final performance measure due to the potential risk of over-fitting to the validation set during the continuous iterations of training-validation experiments.

A more recent study by Ye et al. used a joint CNN-RNN architecture to identify ICH and classify its subtypes^16^. The authors trained their model using both slice-level and subject-level annotations and reported diagnostic accuracy of 99% for ICH detection and accuracy over 80% for categorizing ICH subtypes. Their study shares similarities in the selected DL architecture with the present work. Likewise, the authors used CNN, the de-facto choice for image analysis, for extracting the most valuable features for hemorrhage identification on non-contrast head CT and implemented a bi-directional RNN for aggregating the slice-level predictions of the model. In addition, they implemented the Grad-CAM method to facilitate the interpretation of their models' decisions. However, their study was mainly limited by the relatively low sample size and selection bias. The authors intentionally included CT examinations with hemorrhage to create more balanced datasets as they also admit that their model's performance is yet to be explored in the unselected patient populations^16^.

### Strengths

The present work made several essential contributions to the existing literature on DL-based detection on ICH. First, we used a novel DL architecture, a joint CNN-RNN model with an attention mechanism that shows excellent performance in simultaneously detecting ICH and its subtypes. It has been shown that the attention mechanism allows capturing longer-term dependencies where the performance of standard RNN blocks might be inadequate^23^. To the best of our knowledge, no prior study investigated the utility of the attention method for ICH detection. Second, the black-box nature of the DL is criticized amongst the medical community since it is not always straightforward for medical practitioners to understand the network's decisions. In the present work, we used the NormGrad method, an advancement over its antecedents such as Grad-CAM, and qualitatively showed that NormGrad produces better saliency maps^20^. Third, the lack of prospective external validation in addition to prospective clinical implementation appears to be the core weakness of some earlier studies^12–14,17,18^. We reported the proposed CNN-RNN model's performance with attention mechanism on consecutive unselected patients in a prospective manner in an independent external center. Our results encourage using DL-based methods in the practice for assessing ICH on non-contrast head CT.

### Limitations

Several limitations to this study are needed to be acknowledged. First, we did not compare the model's performance with an average radiologist's assessment of ICH on a head CT scan. The gold standard technique for the ground-truth label is the decision of a radiologist for ICH's presence; thus, we argue that it is to some extent irrational to compare the DL's performance against the gold standard. Nevertheless, several other studies tried to obviate this by using the consensus decisions as the gold standard while using a single radiologist's decisions, preferentially with lesser experience than the gold standard radiologists, as the competitor. Second, we did not incorporate any DL-based segmentation methods to estimate ICH volume in our pipeline. Several prior studies showed the benefits of DL in terms of ICH quantification as quantifying ICH volume is an important yet often neglected task in practice since manually contouring ICH is a labor-intensive and time-consuming operation^30,31^. Third, during the clinical implementation phase, we did not assess whether DL boosted the diagnostic performance or reading time of a radiologist; thus, this is an area of inquiry for future work. Along the same lines, the added value of DL to a radiologist's performance with and without saliency maps should be compared in future studies to justify the value of DL interpretability.

## Conclusions

The joint CNN-RNN model with attention mechanism provided excellent diagnostic accuracy in assessing ICH and its subtypes on a multi-center large-scale sample. The model was seamlessly integrated into the PACS environment and provided its decision within a minute. The pipeline achieved good performance on the test data consisting of consecutive unselected head CT scans obtained in an independent external center for over six months. NormGrad generated saliency maps offer a better model interpretation experience to human radiologists than that of Grad-Cam. Hence, it might be seen as another step towards alleviating the DL's black-box nature in medical imaging tasks.

## Data Availability

Data access requests by qualified researchers trained in human subject confidentiality protocols should be sent to the corresponding author.
